# The role of Open Access Data in democratizing healthcare AI: A pathway to research enhancement, patient well-being and treatment equity in Andalusia, Spain

**DOI:** 10.1371/journal.pdig.0000599

**Published:** 2024-09-16

**Authors:** Álvaro Ritoré, Claudia M. Jiménez, Juan Luis González, Juan Carlos Rejón-Parrilla, Pablo Hervás, Esteban Toro, Carlos Luis Parra-Calderón, Leo Anthony Celi, Isaac Túnez, Miguel Ángel Armengol de la Hoz

**Affiliations:** 1 Big Data Department, PMC, Fundación Progreso y Salud, Seville, Spain; 2 Health Technology Assessment Area, Fundación Progreso y Salud, Seville, Spain; 3 Department of Technology Transfer, Fundación Progreso y Salud, Seville, Spain; 4 Department of Information Systems, Fundación Progreso y Salud, Seville, Spain; 5 Department of Technological Innovation, Virgen del Rocío University Hospital, Seville, Spain; 6 Group of Innovation in Biomedical Informatics, Biomedical Engineering and Health Economics, Institute of Biomedicine of Seville, Seville, Spain; 7 Laboratory for Computational Physiology, Massachusetts Institute of Technology, Cambridge, Massachusetts, United States of America; 8 Department of Medicine, Beth Israel Deaconess Medical Center, Boston, Massachusetts, United States of America; 9 Department of Biostatistics, Harvard T.H. Chan School of Public Health, Boston, Massachusetts, United States of America; 10 Department of Biochemistry and Molecular Biology, University of Córdoba, Córdoba, Spain; 11 Reina Sofía University Hospital, Córdoba, Spain; 12 Maimónides Institute of Biomedical Research of Córdoba, Córdoba, Spain; 13 General Secretariat of Public Health and Research, Development and Innovation in Health, Regional Ministry of Health and Consumer Affairs, Regional Government of Andalusia, Seville, Spain; National Tsing-Hua University: National Tsing Hua University, TAIWAN

Artificial Intelligence (AI), a transformative technology with vast potential in the field of healthcare, presents an array of opportunities for innovation, with the ability to transform medical care from diagnosis to treatment and patient monitoring [1,2]. However, one of the main concerns about AI is the issue of data bias, which refers to the distortion or unfairness that can arise from the data used to train or evaluate AI algorithms. Data bias can affect the accuracy, validity and reliability of algorithms, and can lead to discriminatory or harmful outcomes for certain groups of people [3]. Traditionally, data-driven initiatives have primarily focused on building models and optimizing accuracy, often overlooking the fundamental issue of data bias. This oversight has the potential to propagate algorithmic bias, reinforcing stereotypes and structural inequities, particularly when deployed in real-world scenarios. Thus, it is essential to recognize that many of the models published so far may inadvertently perpetuate disparities present in data sources and patient populations [4].

In the medical field, AI’s most significant contribution lies in illuminating the critical role of data in facilitating more objective, consistent, and immediate decision-making, while promoting health equity [5]. However, healthcare data is powered by the medical knowledge system, which includes influential stakeholders like research funders, universities, researchers, and academic journals. These entities play pivotal roles in determining research priorities, defining patient cohorts, providing platforms for research, and disseminating research findings, whose collective influence significantly shapes the trajectory of medical research and our understanding of health and diseases, thus contributing to the persistence of health disparities [6,7]. In this regard, a significant absence of diversity exists within the medical knowledge system. For instance, more than half of the clinical datasets used for developing and validating AI algorithms originated from either the United States (US) or China [8]. This limited representation of diverse patient cohorts may result in unbalanced model performance globally. Other relevant differences within the AI landscape in clinical medicine include author nationality, sex, clinical specialty, and expertise, further increasing the overall risk of AI bias. Additionally, it’s crucial to underscore the implicit biases among healthcare professionals regarding race/ethnicity, gender, age, weight, socio-economic status, mental illness, or disability, which parallel those found in the general population [9,10]. Unconscious bias towards specific populations may have an influence on clinical decisions, affecting the quality of care provided to patients and perpetuating health disparities across stored medical records, databases, trained models and AI-driven clinical decision-making processes. Overall, a few approaches and frameworks have been considered to reduce AI bias in healthcare [11–13]. Going forward, a paramount concern should be gaining a comprehensive understanding of the underlying data: exploring the composition of patient cohorts, evaluating the accuracy of medical devices across diverse patient groups, or examining monitoring practices in terms of factors like race, sex, socio-economic status or language proficiency. Recognizing and addressing these discrepancies is vital for improving the fairness, reliability, and equity of AI algorithms in healthcare, ultimately bringing the AI-represented world closer to the reality we encounter.

The path to ensure AI benefits everyone may hinge on two crucial requirements. The first entails the establishment of robust regulations and policies shaped through ongoing open dialogue, actively involving communities disproportionately affected by health disparities. The second requirement is to foster an extensive dialogue concerning the methods and stakeholders involved, by shifting the focus from ’what’ to ’who’–those responsible for AI development and deployment–and ’how’–ensuring transparency and accountability in responsible AI practices. In this article, we will explore the ’how’, with a specific focus on achieving transparency and accountability in responsible AI practices, recognizing that building fair and reliable AI algorithms in healthcare necessitates a thorough comprehension of data sources and their inherent biases, while promoting collaboration and knowledge exchange through open-source initiatives. In this context, the utilization of open data emerges as a crucial and potent approach to mitigating data bias within the healthcare domain.

## Open Access Data

In an information-driven world, data has become an extremely valuable resource. The growing demand for access to information has led to a global movement towards openness and democratization of data [14]. In healthcare, Open Access Data refers to data that are available on platforms that foster access to information sets for researchers through a transparent and collaborative process [15]. This collaborative philosophy towards data provides researchers with the opportunity to access and use information relevant to their studies, which drives scientific progress and contributes to knowledge, while promoting the transparency of algorithms and enabling the reproducibility of scientific results by other researchers.

Building fair and reliable AI algorithms in healthcare requires a deeper understanding of the data sources and the potential biases they contain. In this regard, the use of open data represents a very important and effective way to address data bias in healthcare [12,16]. Open data can help detect, correct and prevent data bias by enabling the scrutiny, audit and assessment of algorithms by the scientific, media and civil societies. In addition, open data can also foster innovation and collaboration in the development of more fair, transparent and accountable algorithms that respect ethical principles and human rights. To this end, establishing a robust governance model is essential to ensure secure and ethical handling of open access healthcare data, promoting transparency, reliability, and privacy protection in data-driven research.

### Governance models

To make open access data in healthcare both possible and secure, it is necessary to establish a regulatory and ethical framework that governs its use and protects the privacy of the people whose data are anonymized. In this context, open access data refers to information that is publicly accessible but always subject to rules and standards that regulate its use. Through a rigorous governance process, policies and procedures are defined to safeguard the privacy and security of information, while at the same time promoting transparency and reliability in data access. This governance process involves the multidisciplinary collaboration of various stakeholders, including government agencies, academic institutions, privacy and data security experts, and regulatory bodies. Data use agreements (DUAs) in open access repositories emphasize the user’s commitment to act as a responsible researcher and the need for accountability in data usage. DUAs also state that access to the data should not be shared with third parties and that its use should be limited to legitimate scientific research. Moreover, while the organization sharing the data is legally obligated to take all technically possible measures to prevent patient reidentification, the DUA also requires the user to commit not to attempt patient reidentification. Caution is essential to avoid inadvertently disclosing patient sensitive data in publications and communications, and if any information is found that may allow identification, it should be reported immediately through the established channel to the administrator. Finally, the user is encouraged to share any code associated with publications in a public repository, thus fostering further scientific collaboration. In summary, an operational governance model seeks to establish clear rules on who can access the data, how it can be used, what restrictions can be applied and how patient privacy will be protected. Ultimately, this aims to reduce the time between the request for data and the data analysis process itself.

Two main types of open data can be distinguished based on their level of accessibility: intra-open access data and extra-open access data [17]. These distinctions are based on the accessibility of the data and who can benefit from it. On the one hand, intra-open access data refer to data that are available only to a particular group of users, either limited to a specific geographic region, health center, or research project. This means that only those users belonging to this group have access to the repositories. This category can be useful when dealing with protected health information (PHI) or when there are legal restrictions or policies that limit the disclosure of certain data. Nevertheless, it’s crucial to remember that even within the field of intra-open access data, applying the principles of the aforementioned governance model remains essential. On the other hand, extra-open access data encompasses information that is globally available to the research community. In this regard, researchers worldwide have the opportunity to request access and engage with the data, provided they consent to the DUA and demonstrate a genuine and ethical research purpose. Normally, they should also have a recognized institutional affiliation, a background in health research, and have completed a comprehensive research ethics training course.

For publicly accessible databases, particular attention must be given to patient privacy before data sharing, facilitated by a strong regulatory framework. Health datasets must undergo robust deidentification processes to erase any traces of PHI and to minimize the risk of reidentification. While achieving total data anonymization may not always be possible, and concerns about the security of shared data remain, the drawbacks of hindering healthcare progress through insights from data and restricting medical innovations from the application of AI on publicly available datasets far surpass the risks associated with the potential reidentification of an individual patient [18]. Overall, the European Union (EU) and US differ in their approach to health and data privacy legislation, with the General Data Protection Regulation (GDPR) in the EU and the Health Insurance Portability and Accountability Act (HIPAA) in the US playing central roles [19]. While both aim to ensure robust data protection and provide individuals with control over their data, they vary in scope and approach. Specifically, HIPAA is tailored to the healthcare sector and thus focuses solely on health information, whereas GDPR addresses a wider range of personal data across industries. Consequently, HIPAA’s specificity and less ambiguous provisions, compared to GDPR’s broader approach, facilitates a more clear, direct and precise implementation of security measures, enhancing the protection of health information.

The Open Data philosophy thus presents itself as a catalyst for interdisciplinary collaboration and the creation of integrated solutions that have a real impact on health, by allowing the development of more fair and transparent algorithms, and the reproducibility of results by the scientific community.

### FAIR principles

In the context of publicly available health data repositories, it is important to highlight the FAIR principles (*Findable*, *Accessible*, *Interoperable*, *and Reusable*) that promote the effective and responsible use of data. These principles were developed by a group of experts and international organizations that seek to promote the management and sharing of data in an open and accessible manner [20].

First, data must be "Findable", meaning that they must have clear and descriptive documentation that facilitates their discovery and location. Ultimately, data findability translates into ensuring that resources are easily discovered and accessible by those interested in using them for knowledge generation and informed decision-making. Secondly, data must be "Accessible", which implies that any interested person can interact directly with them. However, when dealing with PHI, additional accessibility restrictions need to be implemented due to the confidential nature of the information. As highlighted earlier, these restrictions may include the need to obtain special authorizations or permissions, to comply with ethical requirements, legal requirements and DUAs, as well as to establish security protocols to protect patient privacy. It is critical that these security measures are implemented in a manner that does not compromise the operability of the data access time. Thirdly, data must be "Interoperable", which means that they must be structured in such a way that they can be combined and used in conjunction with other datasets. This implies the use of common standards and formats that facilitate the integration and exchange of data between different systems and applications. Finally, data must be "Reusable", indicating that they must be prepared and documented in such a way that they can be used in different contexts and by different users. This includes providing information on the terms of use, restrictions and applicable licenses, as well as providing clear and complete documentation that allows the reproduction of the results obtained from the data.

Hence, the FAIR principles offer a sturdy framework that guarantees the ethical and responsible use of publicly available data, all while fostering transparency, collaboration, and research progress. By adhering to these principles, one can bolster data reliability during utilization, facilitate the discovery of novel insights, ease data accessibility, maximize its potential for reuse, and promote the reproducibility of outcomes. This approach, in turn, cultivates a culture of open and shared data to be leveraged by the entire healthcare community for countless medical applications.

### Application of open data in medical contexts

The potential of massive datasets analyzed with Big Data and AI tools is immense and their impact on patient health is going to be paradigm-changing [2]. For instance, during the COVID-19 pandemic, clinical data became a vital resource to improve our understanding of the disease and facilitate more effective decision-making and treatments [21]. Using machine learning techniques and deep learning models, researchers were able to more accurately predict patient outcomes and mortality, opening up new opportunities to deliver personalized and optimized care [22,23]. While some models were primarily used for research purposes, others found practical applications in clinical scenarios. A systematic review identified 66 AI applications that performed a variety of diagnostic, prognostic, and triage functions in the clinical management of COVID-19 [24]. In general, open innovation strategies during global health crises such as the COVID-19 pandemic facilitate an ecosystem for multi-disciplinary relationships, crowdsourcing and accelerated progress and innovation [25]. These advances demonstrate the transformative power of open innovation and open data in healthcare and the tangible benefits they hold for improving people’s quality of life in actual clinical settings.

Open access data repositories in clinical research play a pivotal role in providing a transparent, collaborative and operational platform to address a wide variety of clinical questions. Ultimately, this approach leads to a model of coexistence of different types of repositories, including pseudo-anonymized, anonymized, and synthetic data; these resources further expand research possibilities and promote significant advances in the field of data science in the clinical setting. There has been a significant increase in attention and acknowledgment of open data initiatives within the healthcare sector. In this context, significant international initiatives in the field of healthcare open data are outlined.

*MIMIC* (Medical Information Mart in Intensive Care) is the most widely used clinical database internationally, as a result of collaboration between Beth Israel Deaconess Medical Center and the Massachusetts Institute of Technology. The MIMIC-IV version contains data on approximately 300,000 patients between the years 2008 and 2022, of which more than 70,000 have been admitted to the Intensive Care Unit (ICU) [26].

*eICU* is a collaborative research database populated with data from a combination of multiple critical care units across the continental US. The data in the collaborative database covers patients admitted to critical care units in 2014 and 2015, with information from more than 200,000 ICU admissions [27].

*AMDS* was created by the Amsterdam University Hospital Consortium (Amsterdam UMC) as the first open-access intensive care database within the EU containing deidentified health data related to tens of thousands of admissions to European ICUs, including demographic information, vital signs, laboratory tests, and medications [28].

*The Dutch ICU Data Warehouse* is a project initiated by the Amsterdam University Hospital Consortium (Amsterdam UMC), which comprises severe COVID-19 patient data collected from more than 35 ICUs in the Netherlands [29].

*HiRID* is an open dataset containing information on 33,000 patients admitted to the Department of Intensive Care Medicine of the University Hospital of Bern, Switzerland. This project was promoted collaboratively with the Swiss Federal Institute of Technology (ETH) Zürich [30].

*COVID Data Save Lives* is an anonymized multimodal database comprising medical data from patients treated for the SARS-CoV-2 virus in Spanish private hospitals from the group ’HM Hospitales’. This clinical dataset compiles various interactions within the COVID-19 treatment process, providing comprehensive details on diagnoses, treatments, admissions, ICU visits, diagnostic imaging tests, laboratory results and discharges, among other records [31].

*OPEN DATA COVID* is a secure and anonymized database provided by Spanish healthcare company ’Sanitas’, offering data related to COVID-19 patients admitted to the company’s medical centers for use by the scientific and academic community. This project is part of Sanitas Data4Good, Sanitas’ Open Data initiative that was created with the aim of contributing to society through data, especially in the field of health and well-being [32].

*CARMEN-I* (Corpus of Anonymized Records for Medical information Extraction) is a project created by the Hospital Clínic Barcelona involving the digitalization of medical records. CARMEN-I is designed to be a publicly accessible anonymized health database with the primary goal of advancing technological development and AI applications in healthcare, by providing a structured information format suitable for leveraging Natural Language Processing (NLP) technologies to automatically extract clinical information from the data [33].

These successful international open data initiatives represent a crucial foundation for the development of open-source AI models in healthcare. The applications of AI utilizing open healthcare databases are diverse, encompassing associations between exposures and outcomes [34], predictive modeling [35–37], medical imaging diagnosis [38], clinical decision support systems (CDSS) [39], NLP [40], and reinforcement learning [41]. Moreover, open databases serve as valuable resources for external validation of locally trained algorithms and assessment of performance metrics [42]. The wealth of clinical information amassed in open databases provides a valuable resource for training and fine-tuning AI models. As open datasets continue to grow, so does the potential for open-source AI models to enhance medical diagnosis, treatment personalization, and clinical predictions.

Open access data platforms, both intra and extra, are presented as a guiding philosophy that catalyzes collaboration and the exchange of clinical knowledge, allowing scientists, academics and experts from different areas to work together in the resolution of relevant covered needs. Open data, along with the sharing of source code for analysis, cultivates an environment that encourages collaborations among researchers of various backgrounds and expertise levels, thereby maximizing the potential for new scientific discoveries and breakthroughs. Secure and controlled access to data, coupled with optimized infrastructure and transparency in model training, streamlines the hypothesis validation process and expedites the development of reliable algorithms.

### Open-source AI ecosystem: A beacon of progress in healthcare

The future of AI appears promising, with significant attention being directed towards Large Language Models (LLMs) since the launch of ChatGPT in November 2022 [43]. However, closed-source AI models such as ChatGPT and other LLMs, primarily controlled by Big Tech companies, lack transparency and allow limited accessibility to training datasets and source code. These limitations can result in biased outcomes and perpetuate inequities [4,8]. In response, the open-source AI ecosystem has emerged as an alternative solution, promoting a more accessible, transparent, cost-effective and tunable framework by leveraging the collaborative efforts and ideas generated worldwide [44]. In this scenario, open-source LLMs, such as LLaMA (along with its fine-tuned versions Alpaca, Vicuna, and Wizard), Mistral, or SOLAR, have allowed to drive innovations and advancements in AI and compete with closed-source LLMs [45,46]. Therefore, the development of open-source AI tools emerges as an opportunity for learning and collective development, surpassing the limitations of proprietary models in terms of reproducibility and ethics [47]. In general, this approach fosters a more collaborative mindset for the benefit of patients.

Specifically, several LLM tailored to the healthcare domain have been developed for medical question answering [48], such as *Med-PaLM 2*, a proprietary LLM with medical domain-specific finetuning [49]; *Biomistral*, an open-source LLM using Mistral as foundation model and further pre-trained on PubMed Central [50]; *HuatuoGPT*, an open-source patient-friendly and doctor-like medical advice provider [51]; and *Visual Med-Alpaca*, an open-source biomedical LLM with visual capabilities [52]. Notably, *Med-PaLM* was the first AI model to surpass the pass mark (>60%) in the US Medical Licensing Examination (USMLE) style questions. However, while biomedical-focused LLMs have the innovative potential to democratize medical knowledge and enhance patient care, they still raise concerns regarding patient privacy, validation issues, and ethical aspects such as misinformation and misuse [53]. Bias-related limitations could be mitigated by enhancing the overall LLM development process, including improvements in input data, model architectures, and harmful output detection, along with the promotion of transparent frameworks through publicly available training datasets and open-source code.

Open-source AI technologies applied to healthcare present an opportunity to retrain these large open models for addressing complex tasks in secure health system environments in order to support clinicians in their decision-making. In this context, the use of open healthcare data plays a key role. Through the secure sharing of clinical datasets, the adjustment of these highly customizable models can take place, leading to improved prediction quality and fostering algorithm transparency, collaboration and equity in healthcare. By retraining these models in secure environments, the accuracy and efficacy of diagnostics, personalized treatments and clinical predictions are enhanced, while ensuring equitable access to healthcare and preserving the confidentiality of PHI.

### Leveraging Open Data repositories: Navigating commercialization through data use agreements

While open data repositories offer valuable resources for training algorithms, it’s essential to recognize that open data doesn’t equate to unrestricted commercial use. The nuances of potential commercial use of trained algorithms hinge on DUAs and specific collaboration agreements. Different licenses offer significant commercial freedoms, as detailed in Table 1, which include the Public Domain Dedication and License (PDDL), the MIT License for Data, the Apache License 2.0 for Data, and the Creative Commons License (CC0). However, others, like the Attribution License (ODC-By) or the Open Database License (ODbL), both from Open Data Commons, may impose medium-level restrictions, potentially affecting commercial use. Despite potential restrictions outlined in DUAs and licenses, companies can still derive significant benefits from utilizing open data for research and development, product validation, collaborations, market analysis, and operational efficiency enhancements. Moreover, companies with dedicated research departments can indirectly benefit from open data repositories by entering into specific collaboration agreements with data providers. This collaborative approach fosters knowledge exchange between industry and open data providers, which can pave the way to the collaborative development of innovative solutions tailored to market needs. By adhering to rigorous ethical and security standards, companies can mitigate risks and ensure the reliability of insights gleaned from open data sources.

**Table 1:** Degrees of commercialization freedom in data licensing.

License	Description	Commercialization freedom established by data provider
MIT License for Data	It gives data providers the freedom to commercialize their datasets without any obstacles. It allows them to share their data with others, enabling commercial use, modification, and redistribution, all while ensuring that the provider’s rights are respected. This license empowers data providers to leverage their data for commercial purposes, fostering innovation and collaboration while maintaining a level of flexibility and simplicity in licensing terms.	High
Apache License 2.0 for Data	It enables data providers to freely commercialize their datasets without constraints. It allows unrestricted use, modification, and distribution, fostering collaboration and innovation while safeguarding provider rights.	High
Creative Commons License (CC0)	It grants data providers complete freedom to commercialize their datasets without any restrictions. It allows for the unrestricted use, modification, and distribution of data, fostering collaboration and innovation while safeguarding provider rights.	High
Public Domain Dedication and License (PDDL) de Open Data Commons	It allows data providers to release their data into the public domain, making it available for use without restrictions, including for commercial purposes. This license waives all copyright and related rights that the data provider might have over the dataset, allowing any user to freely use, modify, and share the data.	High
GNU Lesser General Public License for data (LGPL)	Common in software, it can be applied to data. It allows use within proprietary applications if changes to the original LGPL data (or software) are shared under the same license.	Medium
Creative Commons License (CC BY)	It allows use, distribution, modification, and building upon the work commercially, with proper credit to the author.	Medium
Attribution License (ODC-By) from Open Data Commons	It requires attribution when using the data, which may be a minor obstacle, but it does not impose strong restrictions on commercial uses or creating derived products, provided adequate recognition is given.	Medium
Open Database License (ODbL) from Open Data Commons	It is designed to allow users to freely share, modify, and use a database while ensuring that any derivatives remain available under the same license. This license is particularly beneficial from a commercialization perspective because it grants the freedom to use the data commercially, but with the stipulation that any public use of the database, or derivatives thereof, must remain freely available. This ensures that businesses can commercially use and enhance the database, but must keep derivatives open, promoting a cycle of continuous sharing and improvement.	Medium
Customized Licenses	Organizations create their own licenses for specific needs or restrictions on their data. These can be very restrictive, limiting use to non-commercial purposes, prohibiting derived databases without permission, or imposing specific conditions for redistribution.	Low
Creative Commons Attribution-NonCommercial (CC BY-NC)	It explicitly restricts commercial use. However, it’s not typically recommended for software due to its inability to address unique software distribution issues. Nonetheless, some projects use it to limit commercial use or distribution	Low
Aladdin Free Public License (AFPL)	It does not restrict commercial use per se but prohibits the resale of the software. It allows the software to be used in-house within a commercial enterprise. However, it could cause potential legal complications and the likelihood that most software distributions will avoid including programs under these licensesSoftware Engineering Stack Exchange).	Low

## Enhancing the use of healthcare data for research in Andalusia, Spain

With 8.5 million inhabitants, Andalusia is the 30ths most populated region in Europe, surpassing countries like Austria, the Netherlands or Belgium. A significant portion of Andalusia’s population (around 80%), spanning various socioeconomic statuses, relies on public healthcare services [54]. Nevertheless, Andalusia possesses significant potential for clinical research and innovation, given its comprehensive dataset encompassing diverse socioeconomic statuses, health conditions, risk factors, and outcomes across a patient’s medical history spanning more than two decades. Through collaborative analysis of Andalusian data, this resource would serve as critical raw-material for addressing unmet needs identified by healthcare system clinicians and decision-makers, enabling the enhancement of treatments, optimization of diagnostics, and more efficient management of resources.

Through a human and technological infrastructure in massive data processing and AI, the Big Data Department, PMC-Fundación Progreso y Salud (FPS), from the Andalusian Regional Ministry of Health and Consumer Affairs, aims to facilitate the secondary use of Andalusian data for R&I applications in health, clinical practice and management. By employing the philosophy of distributed open collaboration, the resilience of the system in the face of new challenges is improved. To put all this data into value and given the size and complexity of the data, a strategy based on five fundamental principles is being deployed: Security, Integration, Operability, Collaboration and Knowledge. In this regard, ODACI (Andalusian Intensive Care Open Data), PRAETORIA (Andalusian Platform for the Development of Clinical Decision Support Systems) and EVIAS (Assessment and Validation of Artificial Intelligence in Healthcare) stand out as top-notch initiatives and frameworks leveraging the potential of Andalusian healthcare data.

### A potential Andalusian intra-open critical care data initiative, ODACI

ODACI, a Spanish acronym that stands for Andalusian Intensive Care Open Data, is a collaborative open data healthcare initiative from Andalusian ICUs that, if deployed, could improve diagnosis and optimize the treatment of patients through the development of AI algorithms. The source data for ODACI could be Diraya, an Andalusian interoperable electronic medical record storing information from almost 30 hospitals and more than 1,000 primary care centers, integrating data from an accumulated cohort of over 13 million people. ODACI would be governed by the FAIR principles, which guarantee the effective and responsible use of open data in healthcare. Through the opening of ODACI to the scientific community and under strict data protection measures, this clinical database would follow in the footsteps of other similar international open data initiatives that have enabled innovative healthcare improvement through an optimized clinical decision-making procedure based on the application of Big Data and AI analytical tools [55]. ODACI’s initiative has garnered support from the Andalusian Society of Intensive Medicine and Coronary Units (SAMIUC) and the Platform of Patient Organizations (POP). As of May 2024, efforts are primarily directed towards obtaining the necessary authorizations. Being one of the first healthcare data repositories to emerge from the public system, particularly in Andalusia, presents a significant challenge due to the absence of precedents. Ensuring full compliance with legal, ethical, and security regulations is paramount. In line with this approach, ODACI would function as the primary data source for the PRAETORIA platform. This combination perfectly exemplifies how open data drives healthcare innovation, enabling the development of efficient and transparent CDSS within the framework of the PRAETORIA initiative.

### A platform for the development of clinical decision support systems, PRAETORIA

CDSS are technological tools designed to assist healthcare professionals in making informed, evidence-based decisions that promote personalized medicine. These systems use algorithms and computational models to analyze clinical data, such as medical records, diagnostic test results or radiological imaging, with the aim of providing relevant recommendations. By combining medical knowledge and patient information with the power of data processing, CDSS can improve diagnostic accuracy, optimize treatment plans and help prevent medical errors.

In this context, the Andalusian Platform for the Development of Clinical Decision Support Systems (PRAETORIA) has emerged as an opportunity to improve the quality of care, optimize resource management and reduce costs in the Andalusian Public Health System (APHS). PRAETORIA, as an initiative within the APHS, aims to train algorithms using ODACI as data source, which provides accurate and updated patient information, facilitating the process of informed decision-making by healthcare professionals in the diagnosis, treatment and follow-up of patients. Regarding PRAETORIA, several projects are underway to develop CDSS, such as the Horizon Europe-funded project IntelliLung [56], in which Andalusian researchers are participating. Among Andalusian healthcare data projects utilizing CDSS are those focused on combating multi-resistant bacteria and improving healthcare delivery for patients affected by COVID or inflammatory bowel disease, which are in the phase of retrospective validation as of May 2024. Finally, these systems will be evaluated using the EVIAS framework, by addressing concerns about AI-based healthcare technology assessment and patient safety.

### A framework for the validation and assessment of AI in healthcare, EVIAS

The accelerated pace of innovation in the field of digital health technologies and the use of AI pose challenges in terms of decision-making, assessment, and adoption of AI-based healthcare technologies. As AI systems become more common in healthcare, concerns arise about patient safety, generalizability of algorithms, and appropriate interpretation of results by clinicians. In this regard, the EU AI Act, enacted in March 2024, represents the world’s first comprehensive AI law. It aims to ensure safety, protect fundamental rights, and promote innovation in AI technologies. In the healthcare sector, machine learning algorithms are likely to be classified as high-risk AI systems and must comply with specific criteria related to transparency (auditability, bias testing), explainability (data quality, traceability, human oversight), and data governance (data security, data privacy) [57]. Notably, public institutions deploying high-risk AI systems will need to register them in a public EU database. In general, stakeholders will need to adapt to this AI Regulation to foster a more fair, robust, and secure AI ecosystem.

A new framework called EVIAS (Spanish acronym for ’Assessment and Validation of Artificial Intelligence in Healthcare’) is being developed in response to the need to validate and evaluate AI-based health technologies created by both private companies and public research institutions in the healthcare sector. This initiative was promoted by the FPS-Big Data Department in collaboration with the FPS-Health Technology Assessment Area (in Spanish AETSA), and the APHS-Technology Transfer Office (In Spanish OTT). The main objective of EVIAS will be to guarantee the efficacy and added value of AI algorithms, as well as the safety of deploying them in clinical practice. This, in turn, supports institutions in enhancing the quality of their AI development. To achieve this, EVIAS consists of two main phases: the validation of the algorithm using real-world data from the APHS through data science methodologies, and the assessment of AI-based health technologies using Health Technology Assessment (HTA) methods and processes. Consequently, EVIAS introduces a hybrid protocol that combines validation methods utilizing data science tools with assessment criteria from HTA. This new validation/assessment protocol is being carried out in a detailed and comprehensive manner to form a complete framework of procedures and methodologies through the analysis of different healthcare databases initiatives, in order to achieve the best guarantees of efficacy and safety for the algorithms that will support decisions on patient care. As part of this effort, the EVIAS team is reviewing existing HTA approaches for AI-based health technologies, to identify strengths and weaknesses, and develop innovative assessment approaches that go beyond the current state of the art.

Within the ASSESS-DHT project, funded by Horizon Europe and supported by the European Commission, methods required to validate and assess AI-based health technologies are being refined [58]. This collaborative effort involves academic experts, professionals from HTA agencies, independent research organizations, and companies to propose and pilot an assessment framework suitable for digital health technologies, including those leveraging AI algorithms. FPS-based researchers will collaborate with international leaders to address challenges related to validating and assessing AI-based health technologies and other types of digital health technologies. This includes developing real-world validation methods for AI-based algorithms using local data and establishing an HTA framework to evaluate digital health technologies, including AI-based ones. A comprehensive review of protocols endorsed by HTA and regulatory agencies has been conducted, with publication slated throughout 2024.

In summary, the ODACI-PRAETORIA-EVIAS framework embodies an innovative and necessary initiative that can achieve a significant improvement in the quality of care, efficiency in the use of resources and potentially, in some cases, cost reductions. Starting with open healthcare data within ODACI, AI algorithms would be trained and developed within the PRAETORIA platform, facilitating the process of decision-making by healthcare professionals. Finally, these algorithms would be validated and evaluated with EVIAS to ensure trustworthiness, safety and efficacy of prediction models, as well as to assess whether they do actually offer an added value compared to current standard of care. Overall, this workflow promotes the generation of cutting-edge AI tools and valuable knowledge based on open and transparent algorithms, which can be applied in daily medical practice. Ultimately, this open data ecosystem would strengthen the potential for R&D in the field of medicine and position Andalusia as a leader in leveraging data for health and driving significant advances in patient care.

## Conclusion

Open access data represents a dynamic philosophy that not only fosters collaboration and knowledge exchange within the healthcare field but also contributes to the advancement of clinical research, hospital care and patient safety. By granting access to findable, accessible, interoperable and reusable databases, open access promotes scientific progress and the development of fair, transparent and reliable algorithms that drive the reproducibility of results while reducing data bias. Through the implementation of robust governance models and anonymization techniques, privacy risks are mitigated, ensuring that data access meets security, ethics, and compliance standards. The tangible benefits of open data in healthcare research are evidenced by successful international initiatives, which have led to better-informed clinical decisions and enhanced patient outcomes. Moreover, the role of open-source AI in healthcare, particularly the release of open large language models, demonstrates the potential for transformative breakthroughs when open-source collaboration meets advanced technology.

As we look to the future, open data frameworks in healthcare hold the promise of collaborative innovation, personalized medicine and clinical progress. For instance, initiatives like ODACI, PRAETORIA and EVIAS in Andalusia, Spain, exemplify the integration of data-driven clinical decision support systems and AI assessment protocols within a potential open data environment. Through the utilization of open access data and adherence to robust ethical and security standards, these initiatives are poised to drive advancements in diagnostics, treatment optimization and patient safety. In the midst of a rapidly evolving healthcare landscape, the adoption of open access data as the raw material for AI-based health technologies stands as a beacon of progress and health equity, ultimately contributing to a healthier population.
